# *Macrocheles* species (Acari: Macrochelidae) associated with human corpses in Europe

**DOI:** 10.1007/s10493-018-0321-4

**Published:** 2018-11-15

**Authors:** Naila A. Che Kamaruzaman, Peter Mašán, Yelitza Velásquez, Alejandro González-Medina, Anders Lindström, Henk R. Braig, M. Alejandra Perotti

**Affiliations:** 1 Ecology and Evolutionary Biology Section, School of Biological Sciences, University of Reading, RG6 6AS Reading, UK; 2 Institute of Zoology, Slovak Academy of Sciences, 845 06 Bratislava, Slovakia; 3 Department of Environmental Sciences, University of Alicante, 03080 Alicante, Spain; 4 Institute of Legal Medicine of Granada, 18 007 Granada, Spain; 5 Department of Microbiology, National Veterinary Institute SVA, 751 89 Uppsala, Sweden; 6 School of Natural Sciences, Bangor University, LL57 2UW Bangor, Wales, UK

**Keywords:** Shallow grave, Mesostigmata, Acari, Forensic acarology, Decomposition, Carcass

## Abstract

The biology of macrochelid mites might offer new venues for the interpretation of the environmental conditions surrounding human death and decomposition. Three human corpses, one from Sweden and two from Spain, have been analysed for the occurrence of Macrochelidae species. *Macrocheles muscaedomesticae* (Scopoli) females were associated with a corpse that was found in a popular beach area of southeast Spain. Their arrival coincides with the occurrence of one of their major carrier species, the filth fly *Fannia scalaris*, the activity of which peaks during mid-summer. *Macrocheles glaber* (Müller) specimens were collected from a corpse in a shallow grave in a forest in Sweden at the end of summer, concurrent with the arrival of beetles attracted by odours from the corpse. *Macrocheles perglaber* Filipponi and Pegazzano adults were sampled from a corpse found indoors in the rural surroundings of Granada city, south Spain. The phoretic behaviour of this species is similar to that of *M. glaber*, but it is more specific to Scarabaeidae and Geotrupidae dung beetles, most of which favour human faeces. *Macrocheles muscaedomesticae* is known from urban and rural areas and poultry farms, *M. glaber* from outdoors, particularly the countryside, whereas *M. perglaber* is known from outdoor, rural, and remote, potentially mountainous locations. *Macrocheles muscaedomesticae* and *M. perglaber* are reported for the first time from the Iberian Peninsula. This is the first record of *M. perglaber* from human remains.

## Introduction

Mites (Acari) are ubiquitous in the human environment and interact with animals and humans both during life and after death (Braig and Perotti 2009; Goff 1991; Leclercq and Verstraeten 1988a; Perotti 2009; Perotti and Braig 2009a). They inhabit the surrounding environment of any dead body, for instance by living within garments, by nesting in clothing or fabrics, by walking into a corpse or—in the form of phoretic mites—by arriving on a dead body by taking advantage of flying insects and other scavengers for transport (Goff 1991; Perotti and Braig 2009b; Perotti et al. 2010). Despite having been overlooked due to their minute dimensions, mites represent the most diverse eukaryotic organisms of the scavenger community; for each insect species landing on a corpse or a carcass, it is expected that between 1 and 11 + mite species will be carried into the remains (Perotti and Braig 2009b).

That mites occur in human and animal decay is not new. Over 160 years ago, Jean-Pierre Mégnin studied the entomological and acarological fauna of corpses (especially in the morgue of Paris) and already established a sequential colonization of arthropods following stages of decomposition (Braig and Perotti 2009; Leclercq 1978; Mégnin 1894). Within 3 years of the publication of Megnin’s book, Johnston and Villeneueve confirmed the ‘eight waves’ for Canada (Johnston and Villeneuve 1897). The eight waves entered the Manual of Forensic Entomology and became a fundamental part for the understanding of decomposition (Lefebvre and Gaudry 2009; Smith 1986; Wyss and Cherix 2013). The recognition of these waves is very important because it acknowledges that mites in wave 1 are among the first colonizers of a corpse. Mégnin’s wave six, for example, which is associated with a specific stage of decomposition, is exclusively composed of mite species. After Mégnin, it took nearly 100 years until the Belgian pathologist Marcel Leclercq started using mites again in forensic case work to estimate the time of death (Leclercq and Verstraeten 1988a, b, 1993; Leclercq and Watrin 1973). Unfortunately, most of his work was published in French or Dutch and did not reach the English-speaking forensics. Forensic acarology can assist, complement and, at times, even replace forensic entomology (Perotti and Braig 2009a; Perotti et al. 2009). Insects are less likely to colonise corpses during winter months, particularly at high latitudes and altitudes. A corpse that has been covered with lead arsenate as an insecticide and a repellent for police dogs, with the aim of compromising entomological evidence, will still carry forensically important mites (Leclercq and Vaillant 1992). Corpses decomposing indoors or concealed in any other way often carry an abundance of exclusive mites (Frost et al. 2010; Russell et al. 2004; Szelecz et al. 2018). By adding mites to case work evidence, corrections on the insect activity on remains can be made. These include information on insect arrival times, oviposition times, insect life span and departure times—even the end of insect waves can be predicted (Mégnin 1894; Perotti 2009; Perotti et al. 2009, 2010). Used in a similar manner to insects, mites alone can provide timelines too. In this respect, they are of great help in time estimations of later stages of decomposition, when most flesh has disappeared (Mégnin 1894; Perotti 2009; Russell et al. 2004; Saloña-Bordas and Perotti 2014). In addition, mites can become reliable indicators of geographical location or origin (Hani et al. 2018). At genus or species level, mites have micro-habitat specific requirements, offering themselves as potentially one of the most informative pieces of biological trace evidence gathered from a crime scene (Perotti 2009; Pimsler et al. 2016; Prichard et al. 1986; Russell et al. 2004; Szelecz et al. 2018; Webb et al. 1983).

The family Macrochelidae includes over 470 species in 20 genera, and *Macrocheles*, with around 325 described species, is the most diverse genus of the family (Beaulieu et al. 2011; Emberson 2010; Krantz 1962, 1998, 2018; Krantz and Moser 2012; Lindquist et al. 2009; Makarova 2012). New species of macrochelid mites and new phoretic associations are constantly described (Acs et al. 2017; Alatawi et al. 2018; Azevedo et al. 2017; Haloti et al. 2005; Hartini and Dwibadra 2017; Knee 2018; Kontschan 2018; Ozbek 2017). Most *Macrocheles* species are predators feeding on small invertebrates, with the exception of only a handful of non-phoretic, detritivorous species (Manning and Halliday 1994). As predators, they influence population growth of other micro-invertebrates (Geden et al. 1988; Perotti 1999, 2001) and, thereby, may have effects on the advancement and composition of ephemeral micro-ecosystems.

Forensically important *Macrocheles* species arrive on carcasses through phoresy on flies and beetles. Those associated with corpses and carcasses can inform about circumstances of death, environment and habitat, making a link to a site or a location. In a recent crime case study, the inclusion of *Macrocheles matrius* as trace evidence—a species highly prevalent in poultry manure—allowed the reconstruction of the crime scene (Szelecz et al. 2018). Carriers for macrochelids linked to decomposing mammals are necrophagous and necrophilous insects and micro-mammal hosts (Andreev 1988; Halliday 2000; Korn 1983; Krantz and Whitaker 1988; Leclercq and Watrin 1973; Mašán 1999, 2003).

One of the best known macrochelid species, *Macrocheles muscaedomesticae*, is highly prevalent on muscoid flies of the families Muscidae and Fanniidae (Axtell 1964; Filipponi 1960; Perotti and Brasesco 1996, 1997; Rodriguez and Wade 1961; Sacchi Carmona Rodrigueiro and Pires do Prado 2004). These flies colonise and reproduce on a particular variety of organic material (e.g., poultry manure) as well as on sources of food decay abundant in urban areas. They are considered highly synanthropic insects (Legner and Bowen 1973; Perotti 1998). *Macrocheles muscaedomesticae* is much less common on other arthropods and mammals, to a point where it has rarely been reported on Calliphoridae, the dominating fly family of animal decomposition. It can occur on adult blowflies under special circumstances like in indoor decomposition, due to loss of phoretic specificity (Perotti and Braig 2009b). *Macrocheles glaber* and *Macrocheles perglaber* are well known associates of dung beetles (Scarabaeidae, Geotrupidae) (Ciccolani et al. 1981; Filipponi and Pegazzano 1962; Halliday and Holm 1985; Halliday 2000; Mašán 2003; Niogret et al. 2006; Shereef et al. 1990). In Europe, macrochelids on carrion or burying beetles (Silphinae and Nicrophorinae) are outnumbered by species of Parasitidae (Hyatt 1980, 1990; Mašán 1999, 2003). Therefore, any assumptions on phoretic specificity based on unusual or rare reports should be taken cautiously as they might represent a case of loss of phoretic specificity and can compromise the interpretation of the acarological evidence from a crime scene.

Most phoretic macrochelids have a haplodiploid sex determination system termed arrhenotoky, where males are parthenogenetically produced from unfertilized eggs (Manning and Halliday 1994; Norton et al. 1993; Oliver 1977). A few species can be thelytokous and phoretic, like *Macrocheles similis*, a species similar to *M. muscaedomesticae* (Manning and Halliday 1994), and one species, *Macrocheles mycotrupetes*, phoretic on dung beetles, behaves like a diplodiploid (Krantz and Royce 1994). Experiments on arrhenotokous and phoretic *M. glaber* indicated that virgin females can easily be fertilised by their sons, allowing the start of a population (Manning and Halliday 1994). Fertilisation by sons—oedipal reproduction—has experimentally been studied for *M. muscaedomesticae* (Farahi et al. 2018). This is particularly important if the female is a virgin founder. Phoretic *Macrocheles* spp. can travel either as virgin or ‘mated’ females; still, mating does not guarantee fertilisation. The detailed experiments of Costa (1967) on the reproduction of the *Macrocheles pisentii* species complex proposed that wild phoretic females will produce a majority of males in their first progeny, independent of being mated, ruling out a 100% fertilisation. In a new population, as time goes and the number of mites increases, females dominate, to a point where a few males are left in an older dung pad (Kinn and Witcosky 1977; Richards and Richards 1977). This also explains many phoretic females leaving old dung pads unmated or unfertilised.

Recently, confusion has arisen on the matter of the virgin/mated status of phoretic females, with some reports overlooking the fact that founding females will be either virgin or mated—and if mated, they will not necessarily autofertilise their first oocytes (Glida et al. 2003; Kinn and Witcosky 1977; Niogret et al. 2010). Costa (1966) found that slightly old virgin females have difficulties mating, due to the hardening of the genital slits in coxae III, impeding males from the introduction of spermatophora; these females will stay virgin and produce only males, a finding later confirmed by Yasui (1995). Fertilised and unmated *M. muscaedomesticae* will attach to either gender of house flies to move to a new habitat (Jalil and Rodriguez 1970), and the majority of fertilised females will have been exposed in their teneral stage to multiple matings, as males fiercely guard moulting females (Yasui 1995). On the other hand, species such as *M. glaber*, living off the limited habitat offered by an ephemeral (isolated) dung pad, will have difficulties finding mates. Dung-breeding species might resolve sperm competition, sperm precedence and female control on oocyte fertilisation in different ways (Yasui 1995), and might mate just once before departure, if sufficient males occur, otherwise will travel unmated. More research, especially on reproduction of *Macrocheles* species associated with corpses and carcasses, is critical to clarify this phenomenon. Interpreting gender bias of macrochelids would support estimations of time. If the *M. glaber* specimens found in/on a corpse exhibit a male bias, this is suggestive of a recent arrival, of both the carrying beetle and its mites. Under optimal environmental conditions, *Macrocheles* embryos will reach adulthood in just a few (3–4) days (Ciccolani et al. 1977; Singh et al. 1967; Wade and Rodriguez 1961). The sex ratio of the first progeny from a majority of virgin phoretic females (F_1_ generation) will be mainly male-biased, having more males than females, or an even sex ratio within the adult *Macrocheles* population. A few days forward and the sex ratio will transition towards a higher number of females, leading much later towards almost female-only offspring, ready to be transported to a new corpse or carcass, as it happens in nature within dung pads too (Ciccolani 1992).

The biology of three Macrochelidae mite species collected from three corpses decomposing under different environmental conditions is discussed in the light of the potential value these species might offer as indicators of any special circumstances surrounding the death of these individuals.

## Materials and methods

Macrochelidae mites from three case studies occurring in two European countries, Spain and Sweden, were received at Reading University, studied and discussed.

### Case 1

**Fig. 1:**
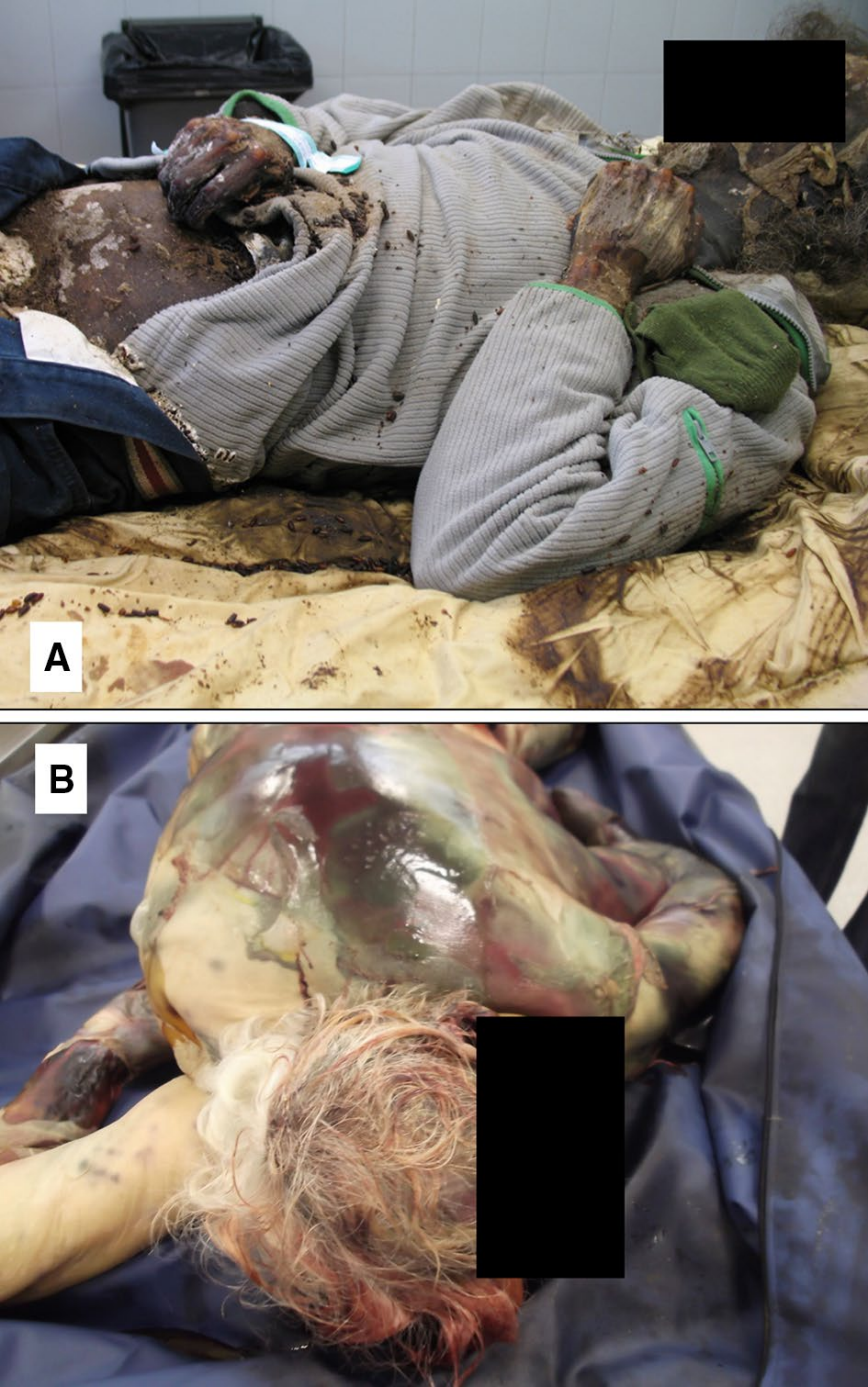
**a** Case 1, the corpse of a homeless man found in a lot close to the beach, Alicante (Spain), in advanced decay and slightly mummified. Photo taken in the autopsy room (YV). **b** Case 3, the body of a woman found in her house in Granada (Spain), in active stage of decomposition. Photo taken in the autopsy room (AGM)

On April 23rd, 2010 (early spring), the corpse of a homeless man was found outdoors, in a lot close to the beach, called ‘Solar Vistahermosa’, Alicante, southeast Spain. The body was found under an umbrella (used for shadow), lying on the ground and face up. It was fully dressed and covered with a blanket up to the neck, exposing only the head. The corpse was reported as in advanced decay, and slightly mummified (Fig. 1a). According to the pathologist, there were no signs of violence and death was stated as natural. The deceased was last seen alive 30 days before the finding. A weather station of the Spanish Meteorological Agency (AEMET) closest to the scene reported an average temperature of 15.3 °C, for the 30 days prior to the discovery of the body.

Entomological evidence was collected from the corpse during autopsy at the Institute of Legal Medicine of Alicante (IMLA), Spain, and consisted of empty puparia and blowfly adults (Calliphoridae: *Calliphora vicina, Chysomya albiceps, Lucilia sericata*); larvae, pupae and empty puparia of *Hydrotaea capensis*; larvae, pupae, empty puparia and adults of *Synthesiomyia nudiseta* (both Muscidae); larvae of the filth fly *Fannia scalaris* (Fanniidae); and pupae of the scuttle flies *Conicera tibialis* and *Puliciphora rufipes* (Phoridae) (Velázquez et al. 2010). The postmortem interval (PMI) was estimated using more than one species of Diptera and gave a maximum of 31 and a minimum of 27 days, which coincided with the time when the person was last seen alive (Velásquez 2011).

Mite samples were prepared for identification at the Acarology Lab (University of Reading) following standards for clearing and mounting of Acari, using Hoyer medium for permanent mounting (Faraji and Bakker 2008). Mites were identified using appropriate taxonomical literature (Emberson 1972; Evans and Browning 1956; Evans and Hyatt 1963; Hyatt and Emberson 1988). Voucher specimens are deposited in the Forensic Acarology Reference Collection, University of Reading.

### Case 2

On September 18th, 2009 (end of summer), the dead body of a woman was found by her boyfriend in a remote forested area in central Sweden. She was reported missing on August 2nd, almost 7 weeks earlier. The area where the remains were found is a boreal forest typical for Sweden with spruce (*Picea abies*), aspen (*Populus tremula*) and birch (*Betula pendula*). The corpse was lying in a very shallow grave, purposely covered with cut aspen branches and birch saplings, grass and moss, revealing only a minor portion of the left hip and right foot. The cover may have delayed the colonization by sarcosaprophagous Diptera for a while but was loose enough to allow colonization by the flies.

The forensic entomologist (AL) visited the crime scene the day after the body’s discovery and sampled insects from the body and nearby surroundings. Another forensic entomology investigation of the scene was held on September 24th when the soil in a radius of approximately 2 m from the center of the grave was dug up to a depth of 15 cm, and collected in search for Calliphoridae pupae. No hatched puparia were found. Adults of *Calliphora vomitoria* started to hatch on September 26th from pupae collected the first time. A time of death was estimated for the first half of August. Due to heavy decomposition of the body, the cause of death could not be established.

Among the entomological specimens collected from the remains, there were many mites. All mite specimens were collected using a brush and transferred to 70% alcohol. Mites were then prepared for identification following the same protocol as for Case 1, and the voucher specimens were deposited in the Reading collection. The identification of the *Macrocheles* species of this case used a variety of keys and descriptions (Halliday 2000; Hyatt and Emberson 1988; Mašán 2003). Mites of the family Parasitidae were also identified (Hyatt 1980).

### Case 3

In September 2011, the dead body of a mature woman, in her mid-fifties, was found in her house in the mountainous country side of Granada, south Spain, in El Sacromonte at an elevation of 820–840 m a.s.l. (González Medina et al. 2012). The discovery was prompted by the odours coming from the house, detected by neighbours. The pathologist determined the cause of death as an overdose of acetaminophen (paracetamol). At the time of the finding, the body was in active decay (Fig. 1b) (indoor decomposition; Galloway et al. 1989; Goff 2009). The deceased suffered from Diogenes syndrome, characterised by self-neglect, isolation, hoarding and accumulation of garbage (González Medina et al. 2012). Insect data were recorded and identified by the forensic entomologist (AGM) and consisted of empty puparia of *Calliphora vicina* (Calliphoridae); adults, larvae and pupae of *Sarcophaga africa, Sarcophaga* sp. (Sarcophagidae); adults of *Musca domestica* and *Hydrotaea aenescens* (Muscidae); adults of *Megaselia* sp. (Phoridae); adults of the clown beetles *Saprinus subnitescens* and *Margarinotus brunneus* (Histeridae); and adults and larvae of the skin beetle *Dermestes frischii* (Dermestidae). According to the original analysis of the case, a PMI of 13 days was estimated based on insect succession and activity of Silphidae and *Poecilochirus austroasiaticus* (Acari: Parasitidae) (González Medina et al. 2012).

A list of mite species associated with the corpse was previously reported, together with an interpretation of the role of the Parasitidae, *P. austroasiaticus* (González Medina et al. 2012). For this study, an unpublished species of Macrochelidae was later rescued from entomological samples of the case, and is discussed here. Mites were kept in 70% alcohol and prepared for identification following the same protocol as for Case 1. Voucher specimens were deposited in the Reading collection. The identification of the *Macrocheles* species of Case 3 followed the description and key of Mašán (2003).

## Results and discussion

Three species of *Macrocheles* were identified, each corresponding to each case study, and the biology of these species was analysed in relation to the corpse and its environmental conditions. Table 1 compiles and expands literature records on habitat and geographic distribution of each species, the table presents a list of phoretic carriers that include common and specific—less-common—species of insects, birds and mammals.

**Table 1:** Literature review and records of the three *Macrocheles* species, with respect to habitat, phoretic carriers and geographic distribution

***Macrocheles muscaedomesticae***						
Habitat						
Animal carcasses		Fresh		Cat	Xero- + mesophytic	Early and Goff (1986), Goff (1989)
				Kangaroo	Grassy woodland	Barton et al. (2014)
		Bloating		Cat	Xero- + mesophytic	Early and Goff (1986); Goff (1989)
		Advanced decay		Kangaroo	Grassy woodland	Barton et al. (2014)
		Skeletal stage		Cat	Xero- + mesophytic	Early and Goff (1986), Goff (1989)
				Impala	Woods	Braack (1986), (1987)
				Bird	?	Emberson (1980)
Human corpses		Fresh			Hospital	Hermann (1804), Oudemans (1929)
		Advanced decay			Small wood	Easton and Smith (1970)
					Near beach	This report
Dung/faeces		Poultry, cattle (outermost layer), pig, wombats (USA: poultry: summer; cattle: winter and spring)				
Bird nests		*Ciconia ciconia, Fulica atra, Larus ridibundus, Merops apiaster, Perdix perdix, Remiz pendulinus, Tachycineta bicolor, Turdus merula, Zapornia tabuensis plumbea*				
Birds		*Dryobates pubescens, Sayornis* sp.				
Mammals		*Apodemus agrarius, Cricetulus barabensis, Eothenomys melanogaster, Homo sapiens, Mus musculus, Myodes glareolus, Notomys alexis, Peromyscus leucopus, Rattus pyctoris, Sigmodon hispidus, Sciurus carolinensis, Spermophilus citellus*				
Reptiles		*Crocodylus johnstoni* (inside mouth), *Terrapene carolina*				
Insect nests		*Reticulitermes flavipes*, bumble bees				
Decomposing plants		Litter				
Other		Facultative parasitism on adult drosophilid and muscoid Diptera				
Phoretic carriers and parasite hosts						
Diptera						
Fanniidae		*Fannia armata, F. canicularis*				
Muscidae		*Australophyra rostrata, Hydrotaea dentipes, Musca domestica, M. sorbens, M. vetustissima, Muscina stabulans, Ophyra chalcogaster, O. ignava, Stomoxys calitrans* (common)				
Calliphoridae		*Calliphora vicina, C. vomitoria, Chrysomya megacephala, Cochliomyia hominivorax, Lucilia cuprina*				
Sphaeroceridae		*Copromyza equina*				
Syrphidae		*Eristalis tenax, Syritta pipiens*				
Coleoptera						
Geotrupidae		*Geotrupes stercorarius*				
Scarabaeidae		*Bubas bubalus, Eupleurus (Aphodius) subterraneus, Catharsius dayacus, Microcopris hidakai, Onthophagus schwaneri, O. waterstradti, Osmoderma eremicola, Scarabeus* sp.				
Silphidae		*Oiceoptoma thoracica, Ptomaphila lacrymosa, Silpha obscura, Thanatophilus rugosus*				
Trogidae						
Distribution						
		[Europe] Arctic (Spitzbergen), Austria, Czech Republic, England, Finland, France, Germany, Greece, Hungary, Italy, Iceland, Poland, Slovakia, Spain (new record), Switzerland, former USSR (Leningrad, Moscow and Stanislav oblasts), Turkey; [Americas] Argentina, Brazil, Ecuador incl. Galapagos islands, Canada, Mexico, USA incl. Hawaii; [Asia] India, Indonesia, Iran, Iraq, Israel, Japan, Malaysia, Philippines, South Korea, Turkey, Tajikistan, Uzbekistan, former USSR (Primorye territory, Sakhalin); [Africa] Egypt, Libya, Saudi Arabia, South Africa; [Oceania] Australia incl. Tasmania, New Zealand				
***Macrocheles glaber*** **sensu lato**						
Habitat						
Animal carcasses		Advanced decay		Fox	Garden	Smith (1975)
				Kangaroo	Grassy woodland	Barton et al. (2014)
Human corpses		Active decay			Forest	This report
					?	Leclercq and Verstraeten (1988a)
Dung/faeces		Chicken, boar, cattle, horse, sheep				
Bird nests		*Accipiter gentilis, Acrocephalus arundinaceus, Anser anser, Ciconia ciconia, Cygnus olor, Larus ridibundus, Merops apiaster, Nycticorax nycticorax, Parus major, P. montanus, Passer montanus, Remiz pendulinus, Vanellus vanellus*				
Mammal nest		Voles				
Decomposing plants		Compost, silage, hay, straw, moss, lichen, bark, rotten wood, seaweed				
Other		Garbage, discarded food				
Phoretic carriers and parasite hosts						
Diptera	Calliphoridae					
	Muscidae		*Australophyra rostrata, Hydrotaea dentipes, Musca domestica, Stomoxys calitrans*			
Coleoptera	Aphodiidae		*Aphodius aestivalis, A. constans, A. erraticus, A. haemorrhoidalis, A. luridus, A. merdarius*			
	Carabidae		*Carabus violaceus*			
	Geotrupidae		*Geotrupes mutator, G. spiniger, G*. (*Anoplotrupes) stercorosus, G. stercorarius, Sericotrupes niger, Trypocopris pyrenaeus, T. vernalis*			
	Histeridae		*Pachylister lutarius*			
	Scarabaeidae		*Aphodius fimetarius, Bubas bison, B. bubalus, Caccobius schreberi, Catharsius molossus, Copris lunaris, Euoniticellus fulvus, Euonthophagus crocatus, Onthophagus coenobita, O. lemur, O. ovatus, O. similis, O. taurus, O. vacca, O. verticicornis, Scarabaeus laticollis, S. sacer*			
	Silphidae		*Nicrophorus humator, N. marginatus, N. obscurus*			
	Staphylinidae					
Distribution						
	[Europe] Belgium, England, France, Hungary, Italy, Latvia, Poland, Slovakia, Sweden, Turkey, former USSR (from the Kola peninsula, Karelia and Yakutin in the north to the Caucasus and Central Asia in the south); [Americas] USA, South America (reported here: considered absent); [Asia] China, Indonesia, Iran, Iraq, former USSR (Central Asia), Taiwan; [Africa] North Africa, Morocco, Réunion, Saudi Arabia; [Oceania] Australia, New Zealand					
***Macrocheles perglaber***						
Habitat						
Human corpse		Bloating—Advanced decay			Indoors	New record, this report
Dung/faeces		Chicken, cattle, horse, sheep				
Decomposing plants		Compost, straw, weeds				
Phoretic carriers and parasite hosts						
Diptera		Muscidae	*Musca domestica, Stomoxys calitrans*			
Coleoptera		Aphodiidae	*Aphodius constans, A. haemorrhoidalis, A. luridus, A. merdarius*			
		Geotrupidae	*Geotrupes mutator, G. spiniger, G. stercorarius, Sericotrupes niger*			
		Scarabaeidae	*Bubas bison, B. bubalus, Copris lunaris, Euoniticellus fulvus, Onthophagus taurus, O. vacca, Scarabaeus cicatricosus, S. laticollis, S. sacer, Sisyphus schaefferi*			
Distribution						
		[Europe] France, Italy, Spain (reported here: new record), Slovakia, Turkey, former USSR (Khabarovsk Territory); [America] USA, South America (reported here: considered absent); [Africa] Morocco, South Africa (reported here: considered absent)				

References specifically assigned to each mite species (this table only)

### Case 1: A dead man found close to a popular beach area, southeast Spain

**Fig. 2:**
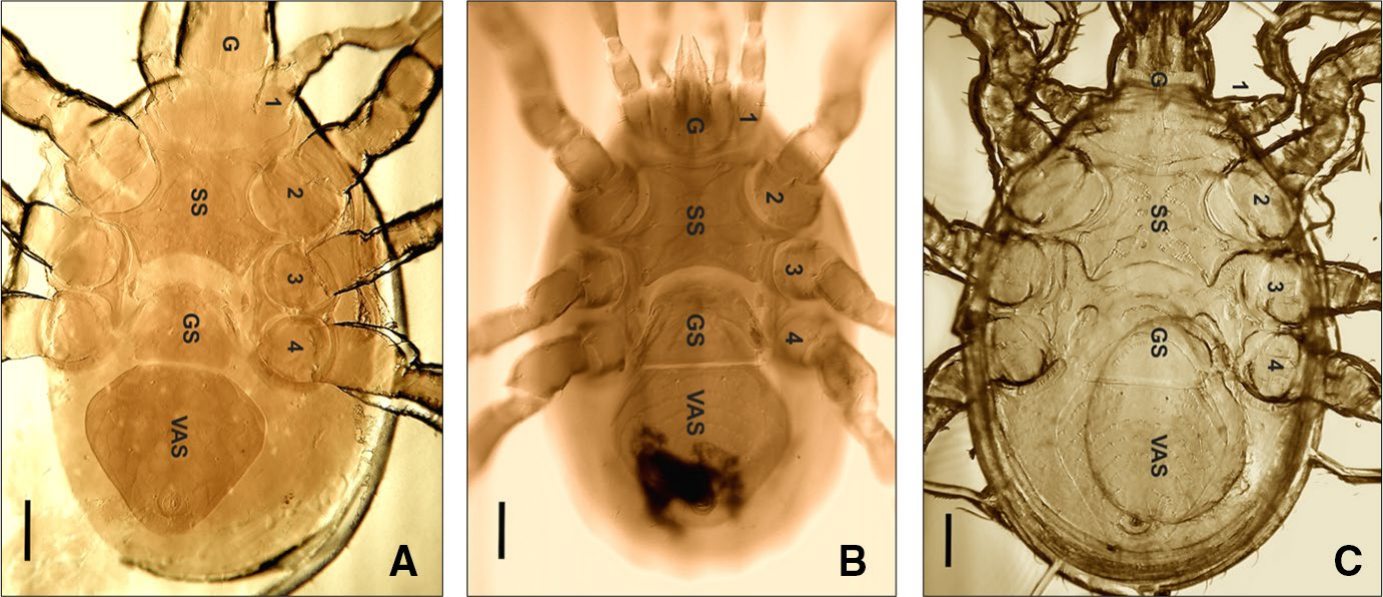
**a** *Macrocheles muscaedomesticae* female, ventral view, identified from the corpse of Case 1 (Spain). **b** *Macrocheles glaber* female, ventral view, identified from corpse of Case 2 (Sweden). **c** *Macrocheles perglaber* female, ventral view, identified from corpse of Case 3 (Spain). Legs are numbered from front to rear; *G* gnathosoma, *SS* sternal shield, *GS* genital shield, *VAS* ventro-anal shield. Scale bars: 100 µm

Two mites were recovered and both were females of *M. muscaedomesticae* (Fig. 2a). *Macrocheles muscaedomesticae* is highly synanthropic, its habitat is domestic, urban and semirural, being common in poultry farms (Farish and Axtell 1971; Ho 1990; Perotti 1996, 1998; Perotti and Brasesco 1996; Rodriguez and Wade 1961; Wade and Rodriguez 1961; Williams and Rogers 1976). It disperses as phoretic on synanthropic animals, preferentially flies (Axtell 1964; Nuorteva 1963) of Muscidae and Fanniidae (filth flies), and much less frequently on other insects or small mammals that live with or in association with humans (Filipponi 1960; Jalil and Rodriguez 1970). Inaccurate identification of mite species riding on insects can lead to confusing reports on phoretic carriers. For example, the latest publication on phoretic mites associated with necrophagous flies in Brazil, reports *M. muscaedomesticae* on the abdomen of *Chrysomya albiceps* (Sato et al. 2018). From the photos included in the publication, disparities emerge from the morphology of the sternal shield of the mites that question the identification of the *Macrocheles* specimens. In fact, none of the mite specimens were identified using keys to species level; instead, the consulted literature were two major keys of Mesostigmata families (methodology section in Sato et al. 2018).

Specific food items of *M. muscaedomesticae* adults are *Musca* and *Fannia* eggs, plus acarid mites. Larvae of *M. muscaedomesticae* feed on conspecifics (cannibalism), and proto- and deutonymphs feed on nematodes (Axtell 1964; Farish and Axtell 1971; Perotti and Brasesco 1996, 1997; Rodrigueiro and do Prado 2004; Rodriguez and Wade 1961; Wade and Rodriguez 1961). Coincidentally, in this case study fly larvae belonging to Muscidae and Fanniidae were collected. *Fannia scalaris* was found at larval stages. This is a highly synanthropic European species associated with food, decay, myiasis, faeces, and with sheltered corpses (Easton and Smith 1970; Leclercq and Verstraeten 1988b; Mégnin 1894; Mihályi 1965; Perotti 1998; Velázquez et al. 2010).

The corpse was sheltered under a beach umbrella and covered by a blanket. Flies and mites independently link the scene of decomposition to a domestic/urban environment. In Europe, there are only two previous reports of *M. muscaedomesticae* on human corpses: (1) mites recovered from the brain, after a failed operation in a military hospital during mid-August in France (of the son of an acarologist), and (2) one female mite, recovered from a human corpse (Easton and Smith 1970; Hermann 1804; Oudemans 1929). The latter was collected together with the Parasitidae species *Poecilochius necrophori*, from a corpse of a poison suicide. The body was found lying on a well-drained chalk hillside, in a small wood on the North Downs in southeast England. This occurred at the beginning of the autumn (October), when Fanniidae and Muscidae flies slow down their activity. Interestingly, according to Easton and Smith (1970), maggots of *Fannia* sp. were collected, although the mite occurred on adult *Musca domestica* (both flies are specific carriers). The body was found in a similar condition to the corpse in the present case. Exposed parts of the body were in advance stage of decomposition, while covered parts (inside a sleeping bag) still had soft tissue and were heavily colonised by the arthropods.

For this case study, *M. muscaedomesticae* adult males were absent and females were rare, possibly due to a late arrival of their carrier flies (likely *F. scalaris*). Indeed, *Macrocheles* first-generation offspring, which is almost exclusively male (Geden et al. 1990; Jalil and Rodriguez 1970) did not complete development. The presence of only two females is in concordance with a minimum number of *F. scalaris* reaching the corpse, as a first generation of flies of the year, early spring, correlate with the moment the corpse was found. *Fannia*’s activity peaks in the summer (Hewitt 1912). Early colonisers, such as Calliphoridae and Muscidae specimens were used for PMI estimations, giving a time since death of approximately 1 month (Velázquez et al. 2010).

Despite being a cosmopolitan species, with most records from European countries, this is the first time *M. muscaedomesticae* is documented within the Iberian Peninsula—possibly due to the lack of work on this taxon in Spain.

### Case 2: A dead woman found in a forest in Sweden

A female (Fig. 2b) and a deutonymph of *M. glaber* sensu lato were collected from the corpse, together with other Mesostigmata mites, mostly Parasitidae deutonymphs known to colonize corpses or carcasses (González Medina et al. 2012; Perotti and Braig 2009a, b; Perotti et al. 2009; Saloña-Bordas and Perotti 2014). *Macrocheles glaber* is the type species of the *glaber* group (Filipponi and Pegazzano 1962), which comprises coprophilous mites associated with large herbivore’s manure, and is less frequently found on carrion (Ciccolani 1992; Fain and Miessen 1997; Filipponi and Pegazzano 1962; Mašán 2003; Perotti and Braig 2009b). It is a cosmopolitan species originally found and studied from the Mediterranean area (Europe and North Africa) (Halliday and Holm 1985; Mašán 2003); however, it has been reported in Sweden from 1998 (Lundqvist 1998; Lundqvist et al. 2000). In a recent survey in Hungary, 224 mites were found in rural forest patches, but only 26 in urban areas, in parks (Mizser et al. 2016). *Macrocheles glaber* is highly prevalent on dung beetles (e.g., Scarabaeidae), occasional on necrophagous and/or necrophilous beetles (e.g., Silphidae) and rare on Diptera (Fain and Miessen 1997; Halliday 2000; Hartini and Takaku 2006; Hyatt and Emberson 1988; Mašán 2003; Mašán and Krištofík 1992; Perotti and Braig 2009b; Perotti et al. 2010). Its phoresy on non-dung-related arthropods (e.g., carrion beetles or filth flies) is assumed as an opportunistic strategy used when its main hosts (dung beetles) are absent (Perotti and Braig 2009b). The corpse was partially covered with local vegetation, that restricted access of beetles and flies. This is not the first report of the species from human remains. Leclercq and Verstraeten (1988a) found *M. glaber* in a body that decomposed during the end of summer and begin of autumn, 3 months after the time of death, with the corpse found in October. Unfortunately, no details of the environment or potential fly or beetle hosts have been provided for this case in Belgium. No common carriers used by *M. glaber* in Sweden are known either (Lundqvist 1998).

*Macrocheles glaber*’s life cycle is slightly longer than that of *M. muscaedomesticae*, completing development after an average of 5 days at 30 °C (females) (Shereef et al. 1990). Like many other macrochelids, the species is haplodiploid, and the F_1_ of female colonisers is mainly male. With sufficient food resources, the female lays eggs that she held under her gnathosoma (oviparity), with poor resources she will lay eggs that hatch immediately (ovoviviparity), and with very poor resources, she will eat her eggs (cannibalism) (Marquardt et al. 2015). It is impossible to sex the deutonymph found but, considering that the accompanying fauna of mites was dominated by Parasitidae (Mesostigmata) and Histiostomatidae (Astigmata), a time of arrival can be drawn (Perotti and Braig 2009b; Perotti et al. 2010; Saloña-Bordas and Perotti 2014). The deutonymph might represent an immature male, offspring of the first females arriving. Seven deutonymphs of *Poecilochirus carabi*, two of *P. mrciaki* (Parasitidae) and three deutonymphs (hypopi) of *Spinanoetus pelznerae* (Histiostomatidae) were recovered from *Necrodes litoralis* sub-elytral cavity, justifying the very recent arrival of the carrion beetle (Silphidae), as much as 2 days before the finding of the body (González Medina et al. 2012). Otherwise, the Parasitidae individuals would have moulted into adulthood. In this sense, the *Macrocheles* specimens have spent long enough on the corpse to produce offspring. If *M. glaber* females arrived earlier, they very likely did on dung beetles. Niogret et al. (2006) carried out a numerical survey of phoront-mite/host species proportions in France and *M. glaber* were highly prevalent on Geotrupidae and Scarabaeidae; proposing that *Aphodius* and *Onthophagus* are the major hosts for the *glaber* group species. Linking this to the geographical location of the case, the most northerly members of the Scarabaeinae are *Onthophagus* beetles with a record of nine species reported for Sweden alone (Ljungberg 2002). The preference of *Onthophagus* for faeces of omnivorous animals, especially human stool, has long been known; some species are also attracted to carrion (Fincher et al. 1970; Howard 1900; Whipple and Hoback 2012; Woodruff 1967). Post-mortem discharge of faeces can occur during fresh decomposition due to relaxing of muscles (*algor mortis*), as well as at the end of the bloating stage, when fluids and excrement exit the body (Shkrum and Ramsay 2007).

In shallow graves, decomposition is delayed and there is no initial scavenger activity (Gaudry 2010; Rodriguez and Bass 1985). In the case study, insect and mite colonization took 6–7 weeks, despite carriers being highly active over the summer. *Macrocheles glaber* has even been recorded in high numbers in Australia at week 6 of decomposition during the summer months (Barton et al. 2014). The Australian study, which recorded a total of 1,003 *M. glaber* from 18 grey Kangaroo carcasses, also recorded very high numbers of beetles in the same week (Barton et al. 2014). Such abundance of *M. glaber* is expected when the mites have arrived earlier, because in this controlled experiment there were no barriers that impeded colonization, the kangaroos were not covered. A small number of mites is expected if there is concealment of the body (as, e.g., in a shallow grave). Analysis of mite numbers should be exercised with caution in a crime scene.

Calliphoridae flies were used in the Swedish case, giving a PMI of 6 weeks, which is supported by the mite evidence.

### Case 3: A dead woman found inside a house in Granada, south Spain

Three Macrochelidae specimens were recovered from the corpse together with other Acari reported elsewhere (González Medina et al. 2012). They were one female (Fig. 2c) and two males of *M. perglaber*. The identification to species level was based on the males, as the morphological differences to females of the sister species *M. glaber* were not conclusive. This has also been the situation for populations from Slovakia (Mašán 2003) and it is expected that many misidentifications of *M. glaber* and *M. perglaber* mites exist in the current literature (Halliday and Holm 1985).

*Macrocheles perglaber* is a member of the *glaber* group, which comprise coprophilous species associated with manure of large herbivores that occasionally are also necrophilous (Ciccolani 1992; Mašán 2003; Perotti and Braig 2009b). *Macrocheles perglaber* presents traits different from *M. glaber. Macrocheles perglaber* has not been found simultaneously with *M. glaber* on the same carriers.

The original population described by Filipponi and Pegazzano (1962) was isolated from horse dung in central Italy. *Macrocheles perglaber*’s phoretic behaviour is similar to that of *M. glaber*, specialising in Scarabaeidae and Geotrupidae (Filipponi and Pegazzano 1962; Glida et al. 2003; Niogret et al. 2010). Most of its dung beetle hosts favour human faeces (Fincher et al. 1970; Howard 1900; Whipple and Hoback 2012; Woodruff 1967). Although *M. perglaber* can be found at any altitude between sea level and 1200–1400 m a.s.l., it seems more restricted to higher elevations compared to its sister species *M. glaber* (Filipponi and Pegazzano 1962; Mašán 2003). The country-side house where the dead body was found is located on a hill (pre-Sierra Nevada Mountains) at an elevation of just over 800 m a.s.l. Livestock is common in this semi-rural area, and considering the waste and abundance of faeces in the house (González Medina et al. 2012), *M. perglaber* was very likely brought inside the house, which had open windows, by scarabs of the surrounding area. According to an inventory of scarabs performed 5 km from the house, at similar altitude, in Pinos de Genil, 12 candidates can be considered: one *Bubas*, five *Onthophagus* and six *Aphodius* species for the pre-Sierra Nevada area (Avila and Pascual 1987). All three genera are attracted to human, horse, cow and other wildlife animal faeces (Woodruff 1967). Death occurred during the summer; therefore, no shortage of potential carriers is presumed.

*Macrocheles perglaber* is haplodiploid, like other macrochelids (Cases 1 and 2). The collection of adult males confirms the presence of the species in the house for at least 5–7 days, as founder females will produce male offspring approximately 5–7 days after arrival to a suitable environment (Kinn and Witcosky 1977; Richards and Richards 1977). The new habitat, the corpse, and the exposition to faeces offered optimal conditions for starting a colony; otherwise, females would have kept attached to the carrier due to their specific requirements regarding the moisture level of the substratum (Niogret et al. 2010). *Macrocheles perglaber* would have arrived with one of the aforementioned dung beetles at an early stage of decomposition, like bloating, which has been confirmed by the presence of *Poecilochirus* mites. Taking into account all these factors, the period of activity of *M. perglaber* in the house would propose a PMI estimation of no less than 8–11 days. This estimation considers (1) the arrival of females at bloating stage, happening 3–4 days after death, and (2) males reaching adulthood in 5–7 days. This minimum period of mite activity agrees with the PMI estimation of 13 days given by the entomological analysis (González Medina et al. 2012).

This is the first report of *M. perglaber* from a human corpse and it is the first report from the Iberian Peninsula.

### Conclusive remarks

The presence of *M. muscaedomesticae* on a corpse, even collected during autopsy, might provide information on the circumstances surrounding death; for example, it may provide links to synanthropic habitats. *Macrocheles glaber* is a mite species transported by beetles, widely prevalent in decomposition. Its specific association with rural environments helps confirming exposure of remains or outdoor decomposition, especially in remote areas. This species cannot access sealed/closed buildings because it rides on large beetle carriers. If found indoors, it is either due to open doors or windows, or due to relocation of the body, from outdoors to indoors. *Macrocheles glaber* is a good indicator of rurality and outdoor habitats including shallow graves. *Macrocheles perglaber* occurrence in outdoor, rural or remote, potentially mountainous, locations is highlighted, due to its specific association with dung beetles. If a corpse is re-located to a new urban location, the presence of this species is indicative of a previous exposure to rural, likely mountainous environment.

## Data Availability

All data generated or analysed during this study are included in this published article.
